# Cytosolic DNA‐STING‐NLRP3 axis is involved in murine acute lung injury induced by lipopolysaccharide

**DOI:** 10.1002/ctm2.228

**Published:** 2020-11-16

**Authors:** Li Ning, Wang Wei, Jiang Wenyang, Xiong Rui, Geng Qing

**Affiliations:** ^1^ Department of Thoracic Surgery Renmin Hospital of Wuhan University Wuhan China

**Keywords:** acute lung injury, cytosolic DNA, NLRP3, sepsis, STING

## Abstract

The role of NOD‐like receptor protein 3 (NLRP3)‐mediated pyroptosis in acute lung injury (ALI) has been well identified previously. Stimulator of interferon genes (STING) is an indispensable adaptor protein, which could regulate inflammation and pyroptosis during infection; however, its role in lipopolysaccharide (LPS)‐induced ALI remains obscure. This study aimed to explore whether STING participated in the development of LPS‐induced ALI as well as the underlying mechanism. We confirmed that LPS significantly enhanced the expression and phosphorylation of STING in lung tissue and primary macrophages from mice. STING deficiency relieved inflammation and oxidative stress in LPS‐treated murine lungs and macrophages. Meanwhile, STING deficiency also abolished the activation of NLRP3 inflammasome and pyroptosis; however, NLRP3 overexpression by adenovirus offset the beneficial effects of STING deficiency in macrophages treated with LPS. Additionally, the level of mitochondrial DNA (mt‐DNA) significantly increased in macrophages after LPS treatment. Intriguingly, although exogenous mt‐DNA stimulation did not influence the level of STING, it could still trigger the phosphorylation of STING as well as pyroptosis, inflammation, and oxidative stress of macrophages. And the adverse effects induced by mt‐DNA could be offset after STING was knocked out. Furthermore, the inhibition of the sensory receptor of cytosolic DNA (cyclic GMP‐AMP synthase, cGAS) also blocked the activation of STING and NLRP3 inflammasome, meanwhile, it alleviated ALI without affecting the expression of STING after LPS challenge. Furthermore, cGAS inhibition also blocked the production of cGAMP induced by LPS, indicating that mt‐DNA and cGAS could activate STING‐NLRP3‐mediated pyroptosis independent of the expression of STING. Finally, we found that LPS upregulated the expression of transcription factor c‐Myc, which subsequently enhanced the activity of STING promoter and promoted its expression without affecting its phosphorylation. Collectively, our study disclosed that LPS could activate STING in a cytosolic DNA‐dependent manner and upregulate the expression of STING in a c‐Myc‐dependent manner, which cooperatively contribute to ALI.

## Graphical abstract

1. Cytosolic DNA‐STING‐NLRP3 axis contributes to LPS‐induced acute lung injury.

2. TLR4 isessential for the activation of cGAS‐STING pathway in macrophages stimulated by LPS.

3. LPS stimulation couldenhance the transcriptional activation of STING via c‐Mycinmacrophages.

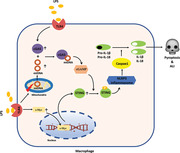

## 1 INTRODUCTION

Sepsis serves as a life‐threatening critical illness caused by dysregulated host responses to infection, which gives rise to approximately 5.3 million deaths each year.^1^ If sepsis is not treated promptly and properly, it may deteriorate into systemic inflammatory response syndrome and multiple organ dysfunction.^2^ Acute lung injury (ALI), serving as one common complication during sepsis, is featured by intractable hypoxemia, impaired gas exchange, severe inflammatory response, and acute respiratory failure.^3^ Due to the complex nature as well as the involvement of multiple cell types (endothelial cells and macrophages), the targeted drug treatment for lipopolysaccharide (LPS)‐induced ALI is absent at present, although the mechanisms contributing to it have been extensively investigated for decades.^4^, ^5^ Additionally, patients surviving ALI are still confronted with long‐term and irreversible physical impairments, severely impacting the quality of life.^6^ It has been recently unveiled that the activation of NOD‐like receptor family pyrin domain containing 3 (NLRP3) inflammasome in macrophages and the production of the proinflammatory cytokines interleukin (IL)‐1β and IL‐18 play a critical role in pyroptosis of macrophages during ALI;^7^, ^8^ however, the regulatory mechanisms of NLRP3 inflammasome in ALI have not been completely addressed.

Stimulator of interferon gene (STING) (also known as MITA, TMEM173, ERIS, and MPYS), acting as a crucial regulator of the DNA sensing pathway, is physiologically embedded in endoplasmic reticulum and could evoke the production of type I interferon by activating nuclear factor‐Κb, as well as interferons regulatory factor 3 pathways.^9^, ^10^, ^11^ In innate immunity, STING is activated by cyclic guanosine monophosphate (GMP)‐adenosine monophosphate (AMP) (cGAMP) synthase (cGAS), which participates in multiple pathological and physiological processes, involving host defense against microbial infections,^12^ cellular senescence,^13^ antitumor immunity,^14^ autoimmunity,^15^ autophagy, as well as many inflammatory diseases.^16^ For instance, in human myeloid cells, targeting the cGAS‐STING‐NLRP3 axis could mitigate inflammasome response during bacterial and viral infections.^17^ Upon HSV‐1 infection, cGAS‐STING pathway could facilitate both the formation and activation of NLRP3 inflammasome.^18^ *Mycobacterium abscessus* infection also gives rise to NLRP3 inflammasome activation of macrophages in a mitochondrial DNA (mt‐DNA)‐dependent manner.^19^ Our recent study unveiled that STING could trigger the activation of NLRP3 inflammasome and pyroptosis of cardiomyocytes challenged with LPS.^20^ These studies point to the possibility that cGAS‐STING pathway may be involved in LPS‐induced ALI by modulating NLRP3 and pyroptosis of macrophage. Therefore, in this study, we aimed to observe whether STING or cGAS deficiency could affect ALI, and meanwhile to uncover the possible mechanisms of STING in ALI.

## 2 MATERIALS AND METHODS

### 2.1 Reagents and antibodies

LPS from *Escherichia coli* O111:B4 purified by phenol extraction (#L2630) and thioglycolate (#T0632) were purchased from Sigma‐Aldrich (St Louis, MO, USA). Primary antibodies against STING (#ab239074), NLRP3 (#ab263899), adaptor apoptosis‐associated speck‐like protein containing CARD (ASC) (#ab175449), Caspase1 (#ab207802 for western blot, #ab138483 for immumohistochemical staining), IL‐18 (#ab243091), Cle‐GSDMD (# ab215203), IgG (#ab133470), 4‐hydroxynonenal (4‐HNE) (#ab46545), and c‐Myc (#ab32072) were obtained from Abcam (Cambridge, UK). Antibodies for IL‐1β (#12703), Phospho‐STING (Ser366), GAPDH (#5174), and cGAS (#83623) were obtained from Cell Signaling Technology (Danvers, MA, USA). Anti‐rabbit/mouse EnVisionTM+/HRP reagent (#GP016129/GP021729) for immunohistochemical staining was bought from Gene Technology (Shanghai, China), while secondary antibody (#926‐32213) used for immunoblotting was obtained from LI‐COR Biosciences. IFN beta ELISA kit (#ab252363) was obtained from Abcam. ROS fluorochrome (#G1045) and AutoFluo quencher were provided by Servicebio (Wuhan, China). The optimum cutting temperature (O.C.T.) compound was purchased from Sakura Finetek (Torrance, CA, USA). Carbonyl Cyanide m‐Chlorophenylhydrazone (CCCP) (≥99.47% purity) (#HY‐100941) and STING agonist‐3 (≥99.94% purity) (#HY‐103665) were acquired from MCE (Shanghai, China).

### 2.2 Animals

All animal procedures in this study were in compliance with the Guide for the Care and Use of Laboratory Animals by the US National Institutes of Health (NIH Publication No. 85‐23, revised in 1996), which were also approved by the Animal Care and Use Committee of Renmin Hospital of Wuhan University (Protocol No. 00013274). STING knockout (C57BL/6J‐Tmem173^gt/J^ *, Sting^gt/gt^*) mice (also known as Golden ticket) with C57BL/6 background (8‐10 weeks old) were acquired from Jackson Laboratory (Sacramento, CA, USA), while cGAS knockout (C57BL/6J‐Cgas^tm1cyagen^, Cgas^–/–^) mice with C57BL/6 background (8‐10 weeks old) were purchased from Model Animal Research Center of Nanjing University. C57BL/6J male mice (8‐10 weeks old) were obtained from the Institute of Laboratory Animal Science, Chinese Academy of Medical Sciences (Beijing, China).^21^ All mice in the present study were kept in a specific‐pathogen‐free environment (temperature 20‐25°C; humidity 50 ± 5%). The LPS‐induced ALI model was established by intratracheal LPS instillation (dissolved in 50 μL sterile saline) at a dose of 5 mg/kg for 12 h as described.^22^ The control groups were given an intratracheal instillation with an isovolumetric sterile saline. After 12 h, the left lung of each mice was excised after mice were sacrificed by cervical dislocation under deep anesthesia. To expand the alveoli, formalin was instilled in the trachea as described.^23^ Then, the lung tissues were dried in an 80°C oven for 48 h until constant weight. The lung wet to dry (W/D) ratio was recorded to assess the condition of pulmonary edema. Survival rate was tracked every 12 h after LPS instillation.

### 2.3 Cell counts in bronchoalveolar lavage fluid

Twelve hours after sterile saline or LPS instillation, the mice were sacrificed and bronchoalveolar lavage fluid (BALF) was collected as described in the previous literature.^24^ In detail, to obtain BALF samples, the murine lungs were lavaged using 1.0 mL ice‐cold phosphate buffer saline (PBS) (pH = 7.4) two to three times. The recovery of the total lavage was over 95%. Then, the BALF samples were centrifuged at 1500 rpm (4°C) for 5 min to pellet the cells, lysed using ACK Lysis Buffer for 3 min, washed three times with ice‐cold PBS, which were centrifuged at 1500 rpm for another 5 min (4°C) one more time. Afterward, the supernatant was stored at –80°C for subsequent measurements. Total cells, macrophages, and neutrophils in BAFL were counted double‐blindly using a hemocytometer, as well as Wright‐Giemsa staining, which were observed under a light microscope (Nikon, Tokyo, Japan, H550L).

### 2.4 Hematoxylin & eosin staining

Hematoxylin & eosin (H&E) staining was performed following previously described.^25^ In brief, the fixed left lung with 10% neutral buffered formalin was embedded in paraffin and transversely sectioned for the observation of inflammatory cells infiltration in pulmonary alveoli.

### 2.5 Immumohistochemical staining

To detect the expression of 4‐HNE and STING, the left lung sections were incubated within xylene for dewaxing followed by gradient ethanol solution to hydrate. 3% H_2_O_2_ was used to make endogenous peroxidase inactivate and 10% goat serum was used to block nonspecific binding sites of proteins. Then, the lung sections were covered with the antibodies against 4‐HNE (1:200 dilution with PBS) and STING (1:100 dilution with PBS) at 4°C overnight. At last, these sections were incubated with an anti‐rabbit EnVisionTM+/HRP reagent at 37°C for 1 h and diaminobenzidine at room temperature for 5 min. Then, the lung tissue sections were photographed under a light microscope (Nikon, H550L).

### 2.6 Determination of oxidative stress and myeloperoxidase activity

The markers of oxidative stress, including malondialdehyde (MDA) (#ab118970) and superoxide dismutase (SOD) (#ab65354), as well as myeloperoxidase (MPO) (#ab25989) in left lung tissues, were measured via commercially available assay kits according to the manufacturer's instructions Abcam website.

### 2.7 Detection of ROS

To begin with, fresh left lung tissues were washed with PBS and then embedded in O.C.T. and snapfrozen in liquid nitrogen as described.^26^ Frozen sections (10 μm) were then cut and mounted on slides and dried before they were used. AutoFluo quencher was covered on the frozen sections of left lung tissue to suppress the autofluorescence. Subsequently, the frozen sections were incubated with ROS fluorescence probe away from light at 37°C for 30 min. Then, cell nucleus was marked using diaminophenyl indole, and the levels of ROS in lung tissues were detected using an Olympus DX51 fluorescence microscope (Tokyo, Japan).

### 2.8 Cell culture and treatment

Mice primary macrophages were isolated and cultured based on the previous description.^22^ To be more specific, macrophages were collected via peritoneal lavage using ice‐cold RPMI 1640 culture medium after the intraperitoneal injection of 3 mL 3% thioglycolate for 3 days. To overexpress NLRP3, macrophages were transfected with Ad‐NLRP3 (MOI = 10) or adenovirus harboring no overexpression sequence (vehicle) for 8 h. The recombinational adenovirus was obtained from HEK293 cells using AdMax system. In detail, the adenovirus was packaged and identified using Opit‐MEM medium. After amplification and purification, the virus titer was detected in HEK293 cells and the expression of NLRP3 was further detected via western blot. To promote the leakage of mt‐DNA in macrophages, CCCP (10 μM) was added to medium for 6 h.^21^ To knockdown the expression of cGAS or toll‐like receptor 4 (TLR4), macrophages were incubated with cGAS siRNA, TLR4 siRNA, as well as the scrambled RNA. Macrophages in our study were transfected using Lipofectamine 3000 (Thermo Fisher Scientific, Waltham, MA, USA) based on the manufacturer's instructions. When the cells reached 75% confluence, macrophages were treated with LPS (100 ng/mL) for 6 h. Experiments were performed at least three times in duplicate.

### 2.9 Reverse transcription, real‑time PCR, and western blot

The total RNA was extracted from the frozen lung tissues or iced macrophages using TRIzol reagent (Invitrogen, 15596‐026) and cDNA was synthesized using a Transcriptor First Strand cDNA Synthesis Kit (04896866001, Roche, Indianapolis, IN, USA). Quantitative real‐time PCR was performed using Light Cycler 480 SYBR Green I Master Mix (Roche, 04707516001) as described.^20^ The expression levels of target genes were uniformly normalized to Gapdh. The primers used in this study are displayed in Table S1.

For western blotting analysis, left lung tissues and cultured cells were homogenized in RIPA lysis buffer, which contained 50 mmol/L Tris‐HCl, 1% Triton X‐100, 0.1% sodium deoxycholate, 5 mmol/L EGTA, 5 mmol/L EDTA, 150 mmol/L NaCl, 40 mmol/L NaF, 2.175 mmol/L sodium orthovanadate, 0.1% SDS, 0.1% aprotinin, and 1 mmol/L phenylmethylsulfonyl fluoride, (pH = 7.2). Then, the tissue or cell homogenate was centrifuged at 12 000 *g* (4°C) for 0.5 h, and the supernatant was collected as protein extracts. Next, a BCA protein assay kit (Thermo Fisher Scientific) was used to measure the protein concentration. After the total proteins of fresh left lung tissues or iced cell lysates were extracted and quantified, approximately 50 μg of total proteins were loaded in an SDS/PAGE gel and were transferred to polvinylidene fluoride membranes. Subsequently, 5% none‐fat dry milk with TBS containing 0.1% Tween‐20 was used to block the nonspecific protein binding sites. After that, the bands containing target proteins were incubated with primary antibodies overnight at 4°C on a shaker. The following day, all bands were washed with PBS three times and incubated with secondary antibodies conjugated to IRDye 800CW for 50 min. Finally, the bands containing target proteins were measured and quantified by the Odyssey infrared imaging system (Odyssey, LI‐COR, Lincoln, NE, USA). Protein expression levels were normalized to the GAPDH internal control.

### 2.10 Determination of IL‐1β and IL‐18 by ELISA

Cell‐culture medium in each group was centrifuged to harvest the cell supernatant. Based on the instructions of the ELISA kits, which were purchased from Abcam, the levels of IL‐1β (ab197742) and IL‐18 (ab216165) in the cell supernatant and lung tissues were detected.

### 2.11 Extraction and detection of mt‐DNA

Mitochondrial DNA Isolation Kit (#ab65321) was purchased from Abcam to extract mt‐DNA in cytoplasm. To begin with, cells were lysed and centrifuged at 700 *g* (4°C) for 10 min. After that, the supernate was collected and centrifuged at 10 000 *g* (4°C) for 30 min. According to differential centrifugation, mt‐DNA in cytosol was in supernate, while mt‐DNA in mitochondria was in sediment. The level of mt‐DNA in cytosol was measured by means of cytochrome C oxidase 1 via real‐time PCR, which was uniformly normalized to 18S. Meanwhile, the mitochondrial lysis buffer and ethanol extraction were used to resuspend sediment, aiming to obtain mtDNA from mitochondria, which was then stored at –20°C. And mt‐DNA was transfected in macrophages using lipofectamine 3000 (L3000015, Thermo Fisher Scientific).

### 2.12 Cell viability

Cell viability is this study was detected using CCK‐8 assay kit (#ab228554, Abcam) as described previously.^27^

### 2.13 Determination of lactate dehydrogenase activity

After primary macrophages were treated with LPS for 6 h, cell culture medium was collected. Based on the standard operating procedure of assay kit, we made up the samples for standard curve using nicotinamide adenine dinucleotide mother liquor and lactate dehydrogenase (LDH) assay buffer. Reaction Mix (50 μL) containing 2 μL of LDH Substrate Mix as well as 48 μL of LDH Assay Buffer was added to standard samples or samples at 37°C for 1 h in the dark. The absorbance was read at 450 nm.

### 2.14 Detection of cGAMP

cGAMP ELISA kit (#501700), which was purchased from Cayman Chemical (Ann Arbor, MI, USA), is a competitive assay for the detection of cGAMP in cell lysates. All samples must be free of organic solvents prior to assay. The detailed procedures were based on the Kit Booklet (https://www.caymanchem.com/product/501700/2%3%-cgamp-elisa-kit).

### 2.15 STING promoter cloning and transcription factor prediction

All STING promoter fragments with various binding sites were cloned into the Kpn I/Bgl II sites in pGL3‐Basic vector via PCR. To identify that the sequence of the plasmids was successfully constructed, DNA sequencing analysis was carried out. Plasmid DNA in this study was prepared using an Endo‐free Plasmid Maxi Kit, which was subsequently transfected as described previously.^28^

Potential transcription factor binding sites were predicted via TFSEARCH (http://www.cbrc.jp/research/db/TFSEARCH.html) software. Then, the mutational primers were designed for these binding sites and site‐specific mutagenesis was performed as reported.^28^ The mutational primers used are displayed in Table S2.

### 2.16 Chromatin immunoprecipitation assay

First, c‐Myc was upregulated via Ad‐c‐Myc transfection in HEK 293 cells. Thirty‐six hours after transfection, the cells were collected and cross‐linked with paraformaldehyde (4%) for 10 min (37°C). Glycine (2 mg/mL) was added to neutralize additional paraformaldehyde followed by cell lysis using an ultrasonic apparatus. Antibodies against c‐Myc and IgG were used for immunoprecipitation. The binding of c‐Myc to STING was detected via real‐time quantitative PCR. The chromatin immunoprecipitation (CHIP) primers used in this study were as follows: F:5′‐GCTCCTACCTAATATCATCCcc‐3′, R: 5‐AGTTATTTCCGGTAACAAGAGC‐3′.

### 2.17 Luciferase assay

To begin with, the promoter of STING (from –519 to +328) was amplified via PCR, which was subsequently identified and separated by 3% agarose gel electrophoresis. The luciferase reporter plasmid STING‐LUC was generated in *E. coli* DH5 competent cells. Subsequently, HEK 293 cells were transfected with a STING plasmid in combination with Ad‐c‐Myc. The luciferase activity was detected and analyzed with the Dual Luciferase Reporter Assay Kit (Promega) based on the manufacturer's instruction.^29^

### 2.18 Statistical analysis

All data in this study were analyzed with the software SPSS 23.3 and were presented as mean ± standard deviation (SD). Two‐way ANOVA followed by a post hoc Tukey test was applied to compare three or more groups, while Student's unpaired *t*‐test was applied to compare two groups. *P*‐value <.05 was regarded to be statistically significant.

## 3 RESULTS

### 3.1 The expression level of STING in ALI

**FIGURE 1:**
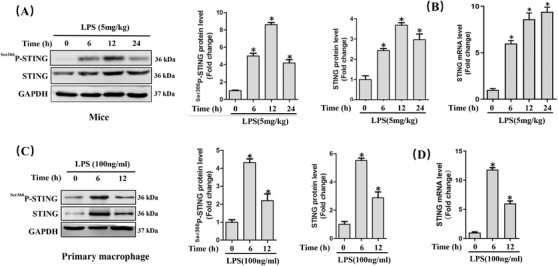
LPS significantly increased the expression of STING in vivo *and* in vitro during ALI. A, Western blots and statistical results in murine lung tissues. B, Relative mRNA level of STING in murine lung tissues after LPS instillation. (n = 6, *P<.05 vs 0 h group). C, Western blots and statistical results in peritoneal macrophages. D, Relative mRNA level of STING in primary macrophages after LPS stimulation (n = 6, *P<.05 vs 0 h group)

First, we observed the expression level of STING in lung tissues from mice challenged with LPS for 0, 6, 12, and 24 h. As shown in Figure 1A, the protein levels of STING and phosphorylated STING at Ser366(^Ser366^P‐STING) were significantly upregulated after LPS stimulation (5 mg/kg). At 12 h, the protein level of STING was at peak level. Real‑time PCR unveiled that the mRNA level of STING increased over time within 24 h after LPS stimulation (Figure 1B). Immunohistochemical staining further confirmed the protein expression of STING in lung tissues (Figure S1). Subsequently, we identified the protein and mRNA expression levels of STING in LPS (100 ng/mL)‐treated primary macrophages for 0, 6, and 12 h in vitro. The results (Figure 1C and D) showed that LPS (100 ng/mL) stimulation for 6 h robustly increased the expression and phosphorylation of STING in primary macrophages. Hence, the time of LPS intervention in mice and macrophages in subsequent experiments was 12 and 6 h, respectively.

### 3.2 STING deficiency attenuated LPS‐induced ALI in mice

**FIGURE 2:**
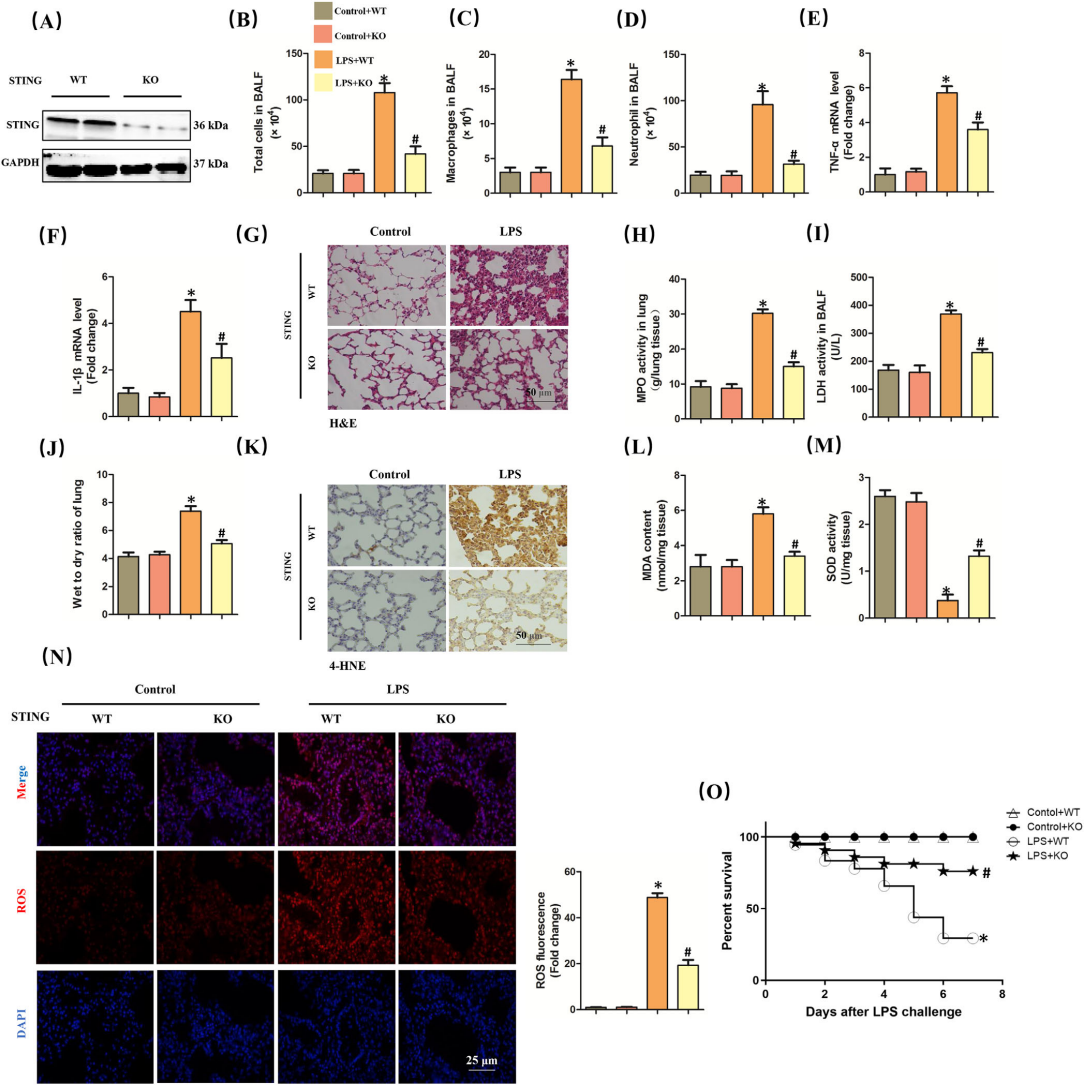
STING deficiency attenuated ALI induced by LPS for 6 h in mice. A, Western blots in murine lung from wild‐type (WT) mice and STING knock out (KO) mice. B‐D, The count of the total cells, macrophages, and neutrophil in BALF. E and F, Relative mRNA levels of TNF‐α and IL‐1β. G, H&E staining for lung tissues. H, MPO activity in lung tissues. I, LDH activity in BALF. J, Wet to dry ratio of lung. K, Immunohistochemistry staining for 4‐HNE protein. L and M, The markers of oxidative stress, including MDA and SOD. N, Representative images of fluorescence probe for ROS in lung tissues (n = 6, *P<.05 vs control+WT group, #*P*<.05 vs LPS+WT group). O, The 7‐day survival rate after LPS instillation (n = 10, *P<.05 vs control+WT group, #*P*<.05 vs LPS+WT group)

To explore the role of STING in LPS‐induced lung injury, the global STING‐deficient mice were used in our study. The protein expression of STING was absent in these lungs from STING‐deficient mice (Figure 2A). STING deficiency significantly prevented LPS‐induced inflammatory response in lung tissues, as indicated by the decreased number of total cells, macrophages, as well as neutrophils in BALF and the reduced mRNA levels of TNF‐α and IL‐1β (Figure 2B‐F). Besides, we also assessed histological alteration of lung tissues via H&E staining. As shown (Figure 2G), LPS significantly promoted the accumulation of inflammatory cells in alveolar; however, the alteration was weakened when STING was absent. Additionally, STING deficiency also attenuated the damage of lung tissue in mice with LPS stimulation, as evidenced by decreased levels of MPO activity in lung tissues, LDH activity in BALF, and lung wet/dry ratio (Figure 2H‐J). Oxidative stress is one of the main features during sepsis‐induced ALI.^30^ Therefore, we next detected the lipid content of peroxidation product (4‐HNE and MDA) and the activity of antioxidase SOD in lung tissues. As expected, LPS gave rise to significant increase of 4‐HNE expression as well as MDA content and significant decrease of SOD activity, which were almost reversed by STING deficiency (Figure 2K‐M). Similarly, STING inhibition also reduced the production of ROS in murine lung challenged with LPS (Figure 2N). What is more, STING deficiency also significantly improves the survival rate of mice with LPS‐induced ALI (Figure 2O). STING deficiency also inhibited the production of type I interferon production (IFN‐β) stimulated by LPS, suggesting STING activation status (Figure S2). Taken together, in this section, we disclosed that STING deficiency could attenuate LPS‐induced ALI in mice.

### 3.3 STING deficiency inhibited NLRP3 inflammasome and pyroptosis in LPS‐treated lung

**FIGURE 3:**
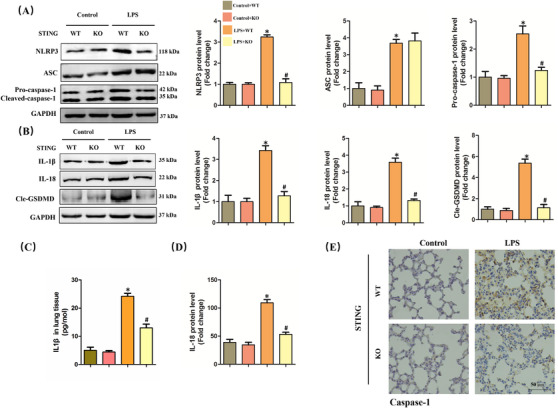
STING deficiency inhibited NLRP3 inflammasome and pyroptosis in lung stimulated by LPS for 6 h. A, Western blots and statistical results for NLRP3, ASC, and Pro‐caspase‐1 in murine lung tissues. B, Western blots and statistical results for IL‐1β, IL‐18, and Cle‐GSDMD in murine lung tissues. C and D, ELISA for IL‐1β and IL‐18 in murine lung tissues. E, Immunohistochemistry staining for Caspase‐1 protein (n = 6, *P<.05 vs control+WT group, #*P*<.05 vs LPS+WT group)

NLRP3 inflammasome‐mediated pyroptosis plays a crucial role in the occurrence and development of ALI.^7^, ^8^ Recently, some studies have unveiled that STING could activate NLRP3 inflammasome and trigger pyroptosis during sepsis.^20^, ^31^ Next, we measured the levels of proteins associated with NLRP3 inflammasome and pyroptosis. The results disclosed that the protein levels of NLRP3 and Pro‐caspase‐1 significantly increased after LPS instillation, which were inhibited when STING was knocked out. It is worth noting that STING deficiency had no effects on the increase of ASC resulted from LPS, hinting that NLRP3 but not ASC served as an effector of STING (Figure 3A). Moreover, we found that STING deficiency also blocked LPS‐induced pyroptosis in lung tissues, evidenced by the decreased protein levels of IL‐1β, IL‐18, and Cle‐GSDMD (Figure 3B). ELISA quantification also showed that STING deficiency significantly inhibited the production of IL‐1β and IL‐18 in LPS‐treated lung tissues (Figure 3C and D). Immumohistochemical staining further showed that the protein level of Caspase‐1 in ALI+STING KO group was significantly lower than that in LPS+STING WT group (Figure 3E).

### 3.4 NLRP3 overexpression abolished the protective roles of STING deficiency in LPS‐treated macrophages

**FIGURE 4:**
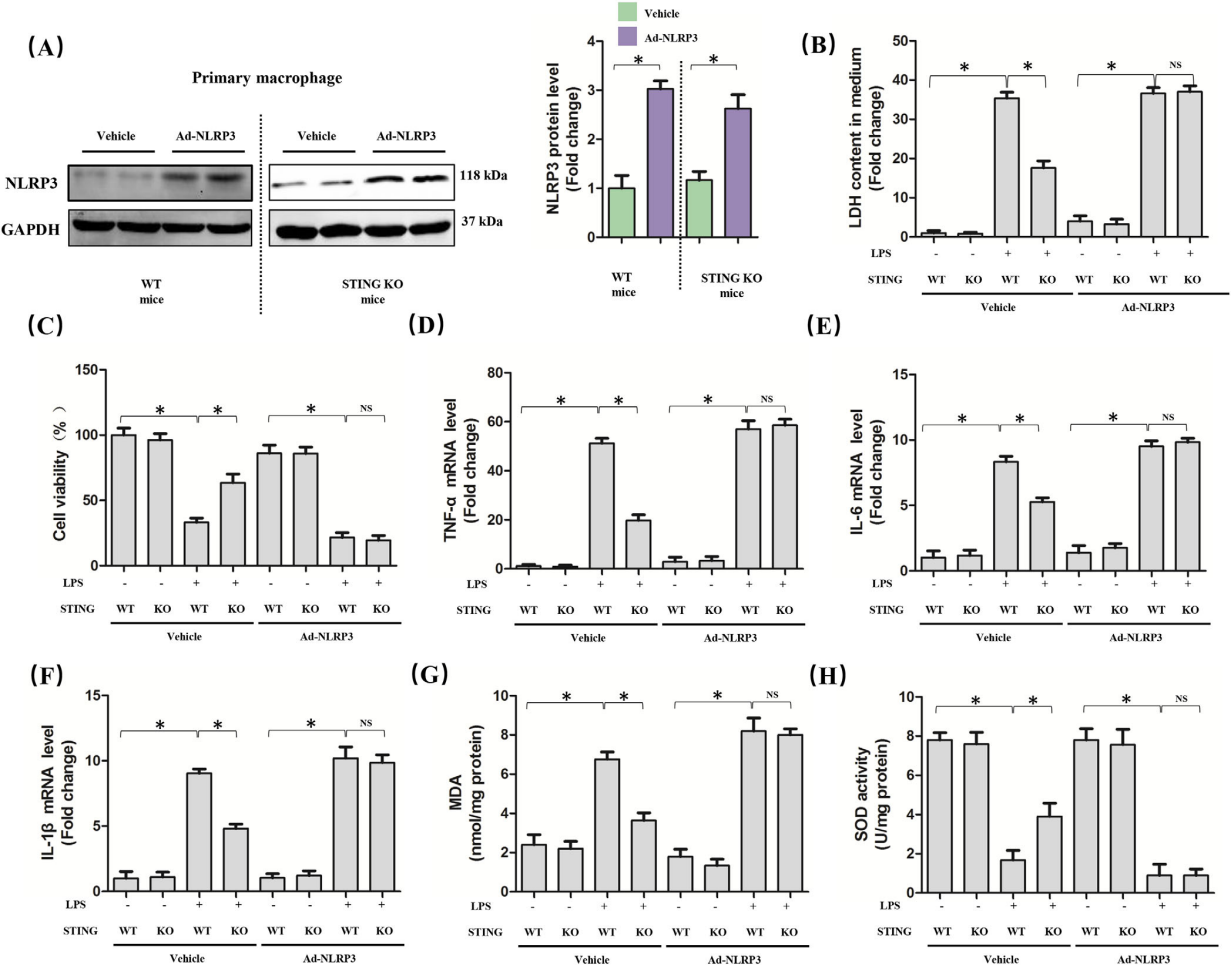
NLRP3 overexpression abolished the protective roles of STING deficiency in macrophages stimulated by LPS for 6 h. A, Western blots and statistical results for NLRP3 in peritoneal macrophages from WT mice and STING KO mice. B, LDH content in medium in indicated group. C, Cell viability was detected by a CCK8 assay in indicated group. D‐F, Relative mRNA levels of TNF‐α, IL‐6, and IL‐1β in indicated group. G and H, The markers of oxidative stress, including MDA and SOD (n = 6, *P<.05 vs indicated group, NS means no significance)

Since STING deficiency could protect against LPS‐induced ALI and inhibit the activation of NLRP3 inflammasome activation, we next explored whether NLRP3 overexpression could offset the protective roles of STING deficiency in macrophages stimulated by LPS. As shown in Figure 4A, we used adenovirus to upregulate the protein levels of NLRP3 in primary macrophages from wild‐type mice and STING‐deficient mice. As expected, STING silencing obviously alleviated cellular damage and increased the cell viability of macrophages stimulated by LPS for 6 h, which was blocked after NLRP3 overexpression (Figure 4B and C). Meanwhile, NLRP3 overexpression also offset the anti‐inflammatory effects of STING silencing in LPS‐treated macrophages, as evidenced by the increased mRNA levels of TNF‐α, IL‐6, and IL‐1β (Figure 4D‐F). Moreover, we also found that NLRP3 overexpression also abolished the antioxidative effects of STING silencing in LPS‐treated macrophages by increasing the level of MDA (Figure 4G) and decreasing the activity of SOD (Figure 4H). These data confirmed that NLRP3 is essential for STING‐mediated cellular damage and inflammation in ALI in vitro.

### 3.5 NLRP3 is essential for STING‐mediated pyroptosis in LPS‐treated macrophages

**FIGURE 5:**
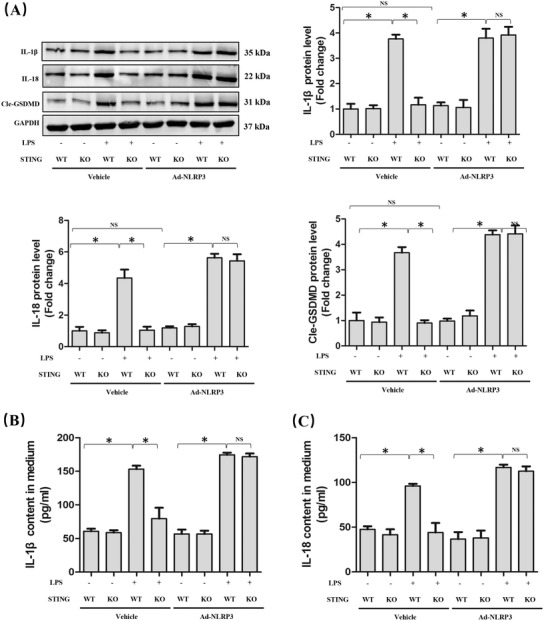
NLRP3 overexpression is involved in STING‐mediated pyroptosis in macrophages stimulated by LPS for 6 h. A, Western blots and statistical results for IL‐1β, IL‐18, and Cle‐GSDMD in peritoneal macrophages. B and C, ELISA for IL‐1β and IL‐18 in peritoneal macrophages (n = 6, *P<.05 vs indicated group, NS means no significance)

Furthermore, we explored whether NLRP3 overexpression could affect STING‐mediated pyroptosis in LPS‐treated macrophages. As expected, NLRP3 overexpression diminished the inhibitory effect of STING deficiency on pyroptosis of macrophages induced by LPS, which was indicated by the increased protein levels of processed IL‐1β, processed IL‐18, and Cle‐GSDMD (Figure 5A). Also, we detected the contents of IL‐1β and IL‐18 in medium from LPS‐treated macrophages, the results of which were in line with their protein expression (Figure 5B and C), indicating that NLRP3 plays an important role in STING‐mediated pyroptosis in LPS‐treated macrophages.

### 3.6 mt‐DNA in cytoplasm triggered NLRP3 activation and pyroptosis in a STING‐dependent manner

**FIGURE 6:**
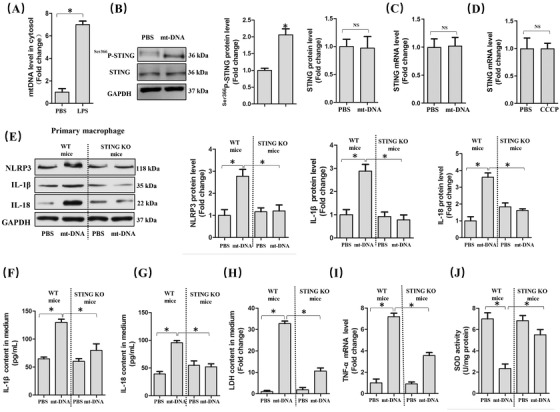
mt‐DNA in cytoplasm triggered NLRP3 activation and pyroptosis in a STING‐dependent manner. A, mt‐DNA in cytosol in peritoneal macrophages. B, Western blots and statistical results for STING and phosphorylated STING in peritoneal macrophages. C, Relative mRNA level of STING in peritoneal macrophages with or without mt‐DNA transfection. D, Relative mRNA levels of STING with or without CCCP pretreatment. E, Western blots and statistical results for NLRP3, IL‐1β, and IL‐18 in peritoneal macrophages from WT mice and STING KO mice with or without mt‐DNA transfection. F and G, Expression levels of pyroptosis markers in culture medium as detected by ELISA. H, LDH content in medium. I, Relative mRNA levels of TNF‐α. J, SOD activity (n = 6, *P<.05 vs indicated group, NS means no significance)

Release of mt‐DNA into the cytosol contributes to the perinuclear translocation and activation of STING, which subsequently enhanced multiple genes associated with inflammation and immune response.^18^, ^32^ LPS could activate the pore‐forming protein GSDMD and form mitochondrial pores, giving rise to the release of mt‐DNA into the cytosol of endothelial cells.^33^ In this study, we observed that LPS stimulation for 6 h significantly increased the level of mt‐DNA in macrophages (Figure 6A). Intriguingly, mt‐DNA from untreated macrophages could not increase the protein and mRNA levels of STING in macrophages, but it could promote the phosphorylation of STING (Figure 6B and C). CCCP could inhibit oxidative phosphorylation and enhance mitochondrial permeability transition, leading to the release of mt‐DNA into the cytosol and cell death.^34^ As expected, CCCP (10 μM) stimulation significantly increased the level of mt‐DNA in cytosol (Figure S3), which had no effects on the mRNA level of STING (Figure 6D). Therefore, we next explored the roles of mt‐DNA and STING on NLRP3 activation as well as cellular damage in vitro. As shown in Figure 6E‐G, mt‐DNA transfection significantly promoted NLRP3‐mediated pyroptosis, which could be reversed by STING deficiency. Additionally, STING deficiency also alleviated cellular injury, inflammation, as well as oxidative stress induced by mt‐DNA transfection (Figure 6H‐J). Thus, we concluded that mt‐DNA in cytosol induced by LPS may contribute to NLRP3 activation and pyroptosis of macrophages in a STING‐dependent manner although mt‐DNA had no effects on the expression level of STING.

### 3.7 cGAS inhibition alleviated ALI in vitro and in vivo

**FIGURE 7:**
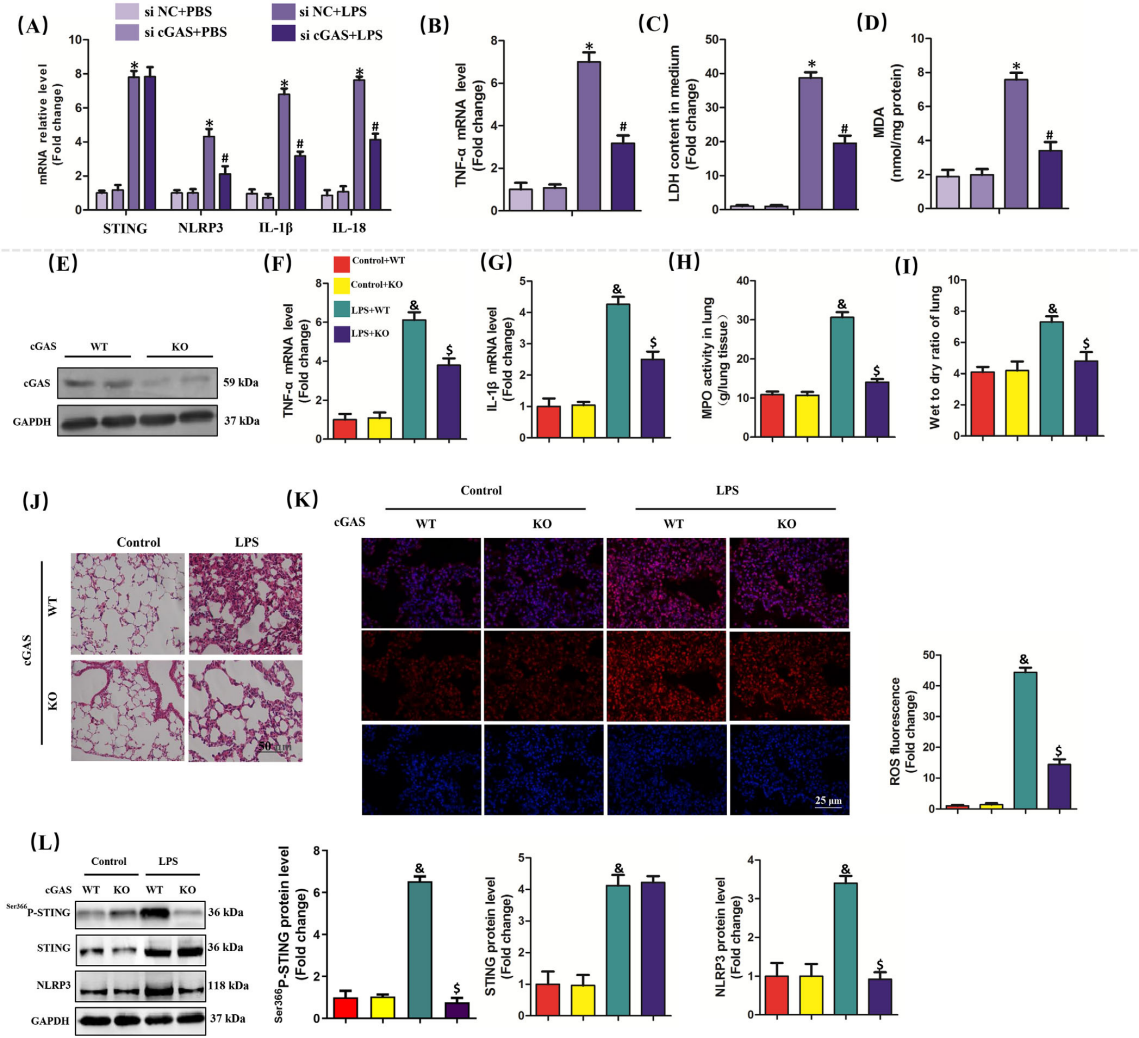
cGAS inhibition alleviated ALI in vitro and in vivo. A, Relative mRNA levels of STING, NLRP3, IL‐1β, and IL‐18 in peritoneal macrophages. B, Relative mRNA level of TNF‐α. C, LDH content in medium. D, MDA content in lung tissues (n = 6, *P<.05 vs si NC+PBS group, #*P*<.05 vs si NC+LPS group). E, Western blots and statistical results for cGAS in lung tissues from WT mice and cGAS KO mice. F and G, Relative mRNA levels of TNF‐α and IL‐1β. H, MPO activity in lung tissues. I, Wet to dry ratio of lung. J, H&E staining for lung tissues. K, Representative images of fluorescence probe for ROS in lung tissues. L, Western blots and statistical results for phosphorylated STING, STING, and NLRP3 (n = 6, ^&^ *P*<.05 vs control+WT group,^$^ *P*<.05 vs LPS+WT group)

cGAS is one of the most‐featured cytoplasmic DNA sensors that could induce cellular injury in mammals and humans.^35^ By binding to cytosolic DNA, cGAS promotes the synthesis of cyclic second messenger GMP‐AMP (cGAMP) through consuming guanosine 50‐triphosphate (GTP) and adenosine 50‐triphosphate (ATP).^36^ Subsequently, cGAMP binds to STING and induces inflammatory injury and the generation of type I interferon via the phosphorylation of STING at Ser366^33^. Here, we found that cGAS silencing significantly blocked the production of cGAMP in LPS‐treated macrophages (Figure S4). cGAS silencing also significantly inhibited the increase of NLRP3, processed IL‐1β, and processed IL‐18 induced by LPS stimulation. However, cGAS inhibition had no effects on the mRNA level of STING, which indicated that cGAS or mt‐DNA is involved in the activation but not expression of STING (Figure 7A). Meanwhile, cGAS silencing also mitigated inflammation, cellular injury, and oxidative stress in LPS‐treated macrophages (Figure 7B‐D). Also, we observed the effects of cGAS deficiency on ALI in vivo using cGAS global knockout mice (Figure 7E). Similarly, cGAS deficiency significantly decreased the levels of proinflammatory cytokines involving TNF‐α and IL‐1β, apart from reducing MPO activity and wet to dry ratio of lung in mice with ALI (Figure 7F‐I). H&E staining also unveiled that cGAS deficiency relieved the accumulation of inflammatory cells in mice with ALI (Figure 7J). Additionally, cGAS deficiency also inhibited LPS‐induced ROS generation in murine lung (Figure 7K). In terms of mechanism, cGAS deficiency significantly inhibited the expression of NLRP3 and phosphorylation of STING induced by LPS; however, it did not alter the protein level of STING in murine lung challenged with LPS (Figure 7L). Additionally, we found that cGAS deficiency could also block LPS‐induced pyroptosis, which was evidenced by the decreased production of IL‐1β and IL‐18 in lung tissues (Figure S5A and B). Collectively, cGAS deficiency could protect against LPS‐induced ALI by inhibiting NLRP3 inflammasome and pyroptosis. Finally, we found that STING overexpression by adenovirus transfection was not sufficient to reproduce the expected phenotype and failed to increase the levels of cytosolic mtDNA (Figure S6A‐D). By contrast, STING activation by agonist could trigger cellular pyroptosis without influencing the levels of cGAS and mtDNA (Figure S6E‐H). These results further proved that there existed axis linking LPS‐mtDNA‐STING‐NLRP3 in a linear fashion.

### 3.8 LPS stimulation enhanced the transcriptional activation of STING via c‐Myc

**FIGURE 8:**
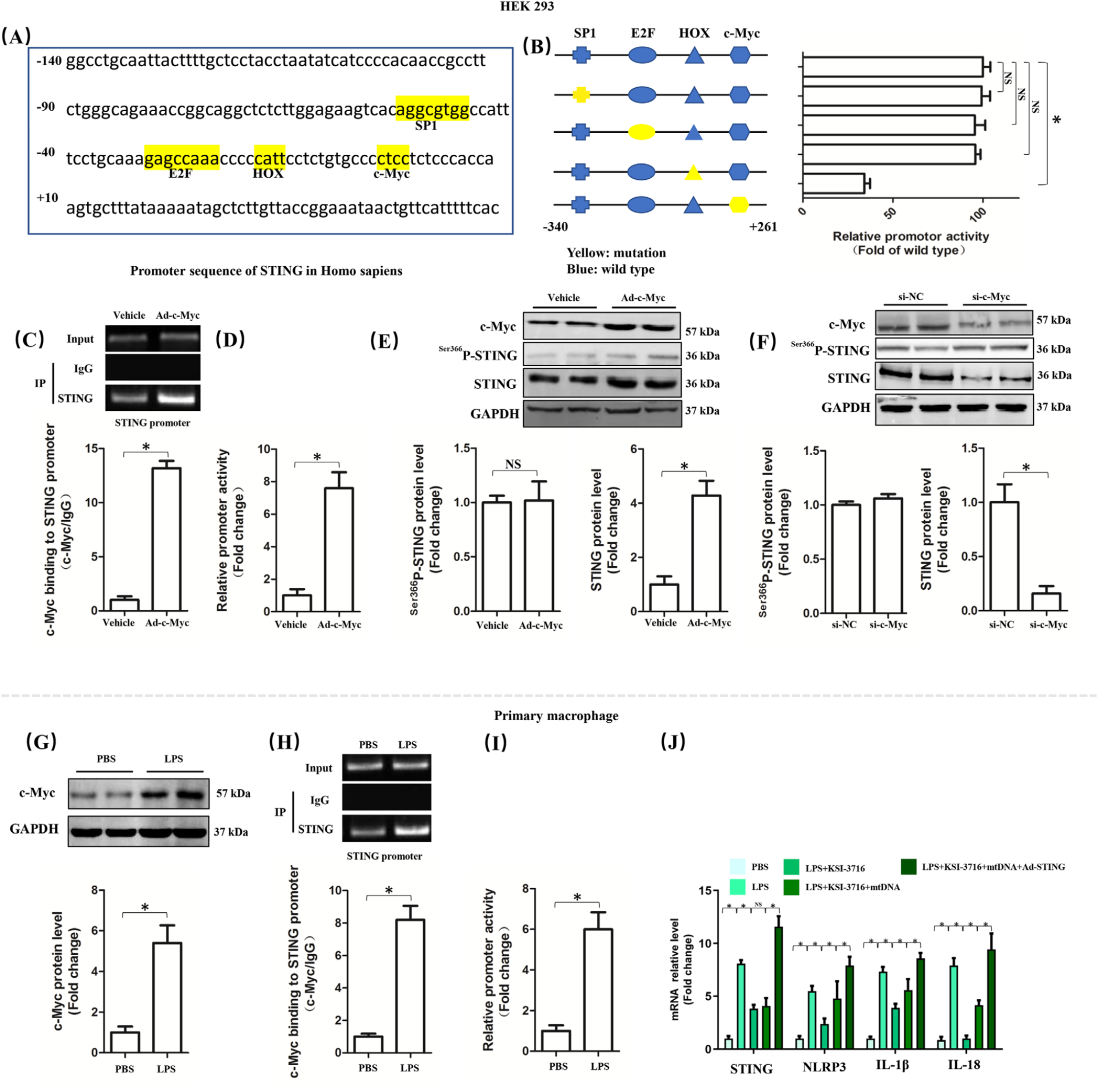
LPS stimulation for 6 h enhanced the transcriptional activation of STING via c‐Myc. A, Promoter sequence of STING in Homo sapiens. B, Mutation analysis of candidate transcription factor for binding sites in promoter region of STING in HEK 293 cells. C, Chromatin immunoprecipitation assay of c‐Myc and STING promoter with or without c‐Myc overexpression in HEK 293 cells. D, Relative promoter activity of STING with or without c‐Myc overexpression in HEK 293 cells. E and F, Western blots and statistical results for phosphorylated STING and STING after c‐Myc was upregulated or inhibited. G, Western blots and statistical results for c‐Myc with or without LPS stimulation in peritoneal macrophages. H, Chromatin immunoprecipitation assay of c‐Myc and STING promoter. I, Relative promoter activity of STING. J, Relative mRNA levels of STING, NLRP3, IL‐1β, and IL‐18 (n = 6, *P<.05 vs indicated group, NS means no significance)

Since mt‐DNA or cGAS was responsible for the activation of STING, LPS may upregulate the expression of STING by other mechanisms. Hence, we next predicted the transcription factor binding sites of STING of HEK 293 cells via bioinformatics analysis. Core functional area of STING promoter was between –120/+10 bp, where existed the potential candidate binding sites of SP1, E2F, HOX, and c‐Myc (Figure 8A). Mutation of binding sites of SP1, E2F, HOX, and c‐Myc further disclosed that mutation of binding sites of c‐Myc but not SP1, E2F, or HOX could significantly reduce the promoter activity of STING (Figure 8B). The results of CHIP showed that overexpression of c‐Myc by adenovirus in HEK 293 cells could enhance the binding of c‐Myc to STING promoter (Figure 8C), in addition to increasing the promoter activity of STING (Figure 8D). What is more, overexpression or inhibition of c‐Myc significantly increased or decreased the protein level of STING without affecting its phosphorylation (Figure 8E and F), suggesting that c‐Myc played a vital role in the expression of STING. To validate our hypothesis, we also detected the protein level of c‐Myc in LPS‐treated macrophages. As shown in Figure 8G, LPS stimulation enhanced the protein expression of STING in macrophages. Meanwhile, the binding of c‐Myc to STING promoter, as well as the promoter activity of STING in macrophages, was also enhanced in LPS‐treated macrophages (Figure 8H and I). At last, we used KSI‐3716, an inhibitor of c‐Myc, to further identify the role of c‐Myc in STING‐mediated pyroptosis in macrophages challenged with LPS. The results (Figure 8J) showed that KSI‐3716 pretreatment could inhibit the mRNA levels of STING, NLRP3, IL‐1β, and IL‐18 in LPS‐treated macrophages; however, mt‐DNA significantly decreased the mRNA levels of NLRP3, IL‐1β, and IL‐18 except STING. By contrast, overexpression of STING reversed the effects of KSI‐3716 in combination with mt‐DNA in LPS‐treated macrophages, which further proved that LPS could enhance the activity of STING in a mt‐DNA‐dependent manner and its expression in a c‐Myc‐dependent manner, respectively. Taken together, the increase of c‐Myc and mt‐DNA induced by LPS cooperatively gave rise to pyroptosis of macrophages. Since LPS serves as a classical activator of TLR4, the expression of TLR4 in macrophages was knocked down by TLR4 siRNA. The results (Figure S7) demonstrated that TLR4 inhibition in macrophages counteracted the upregulation of cGAS, STING, and c‐Myc triggered by LPS, indicating that TLR4 was essential for the activation and expression of STING.

## 4 DISCUSSION

In this study, we found that LPS treatment promoted the activation and expression of cGAS‐STING‐NLRP3 axis of macrophages and cGAS or STING deficiency significantly alleviated LPS‐induced ALI of mice by inhibiting inflammation, oxidative stress, and cellular injury. Mechanistically, we identified that cGAS or STING deficiency could relieve NLRP3‐mediated pyroptosis of macrophages. Moreover, we found that LPS increased the level of mt‐DNA in cytosol, which further gave rise to the upregulation of cGAS and the activation of STING. Meantime, LPS also upregulated the expression of c‐Myc, which served as a transcription factor of STING and promoted its expression. On the basis of these findings, we supposed that cytosolic DNA‐STING‐NLRP3 axis was a possible therapeutic target against LPS‐induced ALI.

Macrophages are the most abundant immune cells in lung tissues, owing to the critical task of maintaining tissue homeostasis by initiating host defense, upon pathogens stimulation.^37^ Timely suppression of inflammatory signaling pathway of macrophages is critical for balance of tissue homeostasis, imbalance of which gives rise to ALI because of the accumulation of leukocytes as well as protein‐rich fluid in the alveolar space.^38^ Previous studies have demonstrated that NLRP3 inflammasome activation in macrophages is involved in the inflammatory responses of ALI, the inhibition of which may lead to the alleviation of ALI.^8^, ^39^ LPS stimulation could cause the generation of ROS and ASC induction in lung tissues, which binds to NLRP3, giving rise to NLRP3 inflammasome activation. Subsequently, inflammatory cascade was activated and amplified, leading to the release of mature IL‐1β as well as IL‐18 and cell death.^40^

STING, serving as a transmembrane homodimer of the endoplasmic reticulum membrane, could potently induce macrophage inflammatory signaling during lung injury. For instance, monocyte‐derived macrophages, which are recruited into the airspace, could enhance the anti‐inflammatory function of macrophages by blocking their STING signaling.^41^ STING also contributes to bleomycin‐induced idiopathic pulmonary fibrosis and lung injury, which could be alleviated by juglanin.^42^ Additionally, acute exposure to cigarette smoke also causes airway inflammation with fibrosis and emphysema of mice in a STING‐dependent manner.^43^ Our recent study proposed that STING may drive NLRP3 inflammasome‐mediated pyroptosis and cellular injury in sepsis‐induced cardiac injury.^20^ During liver ischemia and reperfusion, STING suppression of macrophages inhibited the overactivation of NLRP3 inflammasome and excessive secretion of IL‐1β and IL‐18, eventually reducing liver injury.^44^ However, to our knowledge, there is still lack of explicit data on the role of STING in ALI induced by LPS. In the present study, we discovered that STING deficiency in vivo and in vitro could relieve LPS‐induced cellular injury, inflammation, oxidative stress, and pyroptosis in macrophages; however, the beneficial effects from STING deficiency were abolished after NLRP3 overexpression, indicating that the proinflammatory roles of STING relied on the activation of NLRP3.

Although DNA carries large amounts of genetic information, “mislocalized” self‐DNA can also induce endogenous inflammation. Previous studies have unveiled that self‐DNA sensing by the immune system acts as a crucial contributing response in various lung inflammatory diseases, including emphysema, chronic obstructive pulmonary disease, interstitial lung diseases, as well as idiopathic pulmonary fibrosis, which are all featured by cell stress as well as death with DNA release. Once mitochondrial stress occurs, activation of the mitochondrial permeability transition pore may give rise to the leakage of linear mt‐DNA fragments into the cytosol and the activated DNA sensor cGAS, triggering STING signaling. LPS has been proved to initiate mt‐DNA damage by oxidative mechanisms.^45^, ^46^ In this study, we observed that LPS or CCCP stimulation significantly increased the level of mt‐DNA in cytosol; however, the expression level of STING was not affected after mt‐DNA transfection or CCCP pretreatment although mt‐DNA could induce NLRP3‐mediated pyroptosis of macrophages in a STING‐dependent manner. We also found that LPS promoted the production of cGAMP, indicating that the increased mt‐DNA induced by LPS was responsible for STING activation.

The evolutionarily conserved c‐Myc is a member belonging to proto‐oncogenic transcription factors family, which is closely involved in proliferation, apoptosis, and growth of cancer cells. Studies have also shown that the inhibition of c‐Myc could give rise to a suppression in proinflammatory signaling. Zhang et al^47^ reported that MCP‐induced protein 1 could relieve LPS‐induced ALI and inflammation by modulating c‐Myc‐mediated macrophage polarization. Our study demonstrated that c‐Myc was upregulated in macrophages during ALI, and c‐Myc served as a transcription factor of STING and was responsible for transcriptional activation by directly binding to promoter of STING and enhancing promoter activity, indicating that c‐Myc was also involved in the transcription of genes associated with innate immunity and inflammation. It is worth noting that upregulating c‐Myc under basal conditions may also directly promote the expression of STING.

In conclusion, we found that mt‐DNA in cytosol and c‐Myc cooperatively activate and upregulate STING, which subsequently leads to LPS‐induced ALI by promoting NLRP3 inflammasome and pyroptosis of macrophages. Our study has provided promising evidence for the targeting of STING‐NLRP3 against ALI and sepsis.

## CONFLICTS OF INTEREST

The authors declare no conflicts of interest.

## AUTHORS’ CONTRIBUTION

Li Ning, Wang Wei, and Geng Qing contributed to conception, designed experiments, and took full responsibility for the whole work; Li Ning, Jiang Wenyang, and Xiong Rui performed experiments; Li Ning and Wang Wei analyzed experimental results; and Li Ning and Jiang Wenyang wrote the manuscript.

## Supporting information

Supplementary Material

## Data Availability

All data that support the findings in this study are available from the corresponding author upon reasonable request.
