# Supplementary material for: Cytosolic DNA‐STING‐NLRP3 axis is involved in murine acute lung injury induced by lipopolysaccharide
Source: Clin Transl Med. 2020 Nov 16;10(7):e228. doi: 10.1002/ctm2.228 (PMC7668192; doi:10.1002/ctm2.228)

**Supplementary Table1** The primers used in real-time PCR

| **Species** | **Gene** | **Forward primer** | **Reverse primer** |
| --- | --- | --- | --- |
| Mice | *Sting* | CACGCGTCCGCCCAC | TCGAGACTCGGGGACATCTT |
| Mice | *Tnf-α* | ACTGAACTTCGGGGTGATCGGT | TGGTTTGCTACGACGTGGGCTA |
| Mice | *Il-18* | GTTTACAAGCATCCAGGCACA | TCATGCAGCCTCGGGTATTC |
| Mice | *Il-6* | CCCCAATTTCCAATGCTCTCC | CGCACTAGGTTTGCCGAGTA |
| Mice | *Il-1β* | AATGAAGGAACGGAGGAGCC | CTCCAGCCAAGCTTCCTTGT |
| Mice | *Nlrp3* | CCAGAAACTGTGACTGTACATTGG | TTGGTCCCACACAAGCCTTT |
| Mice | *Caspase1* | GAAACGCCATGGCTGACAAG | ACTTGAGGGTCCCAGTCAGT |
| Mice | *Gapdh* | ACTCCACTCACGGCAAATTC | TCTCCTATGGTGGTGACGACA |
| Mice | *Cytochrome C oxidase 1* | ATTGTCGATGATGCTGATGC | CTGTCGTAGTCGTAGTGTCA |
| Mice | *18S* | AGCTGATGTCGTAGTCGTGAT | CTAGTGTGTAACGTAACG |

**Supplementary Table2** The mutational primers for different binding sites of STING promoter

| **Species** | **Gene** | **Forward primer** | **Reverse primer** |
| --- | --- | --- | --- |
| Human | *Sp1-m* | GAAATGGCCAGAACTGTGACTTCTCCAAGA | CTGCAAAGAGCCAAACCCCCATTCCTCT |
| Human | *E2f-m* | CCCCCATTCCTCTGTGCCCcTCCTCT | TTTGCAGGAAATGGCCACGCCTGTGACTT |
| Human | *Hox-m* | GGGGTTTGGCTCTTTGCAGGAAATGGCCA | TCTGTGCCCCTCCTCTCCCACCAAGTG |
| Human | *c-Myc-m* | CTCTCCCACCAAGTGCTTTATAAAAATAGCTC | GGCACAGAGGAATGGGGGTTTGGCT |

**Figure legends**

**Supplementary Fig. 1** Immunohistochemistry staining for STING protein in lung tissues instillation with or without LPS.

**Supplementary Fig. 2** The role of STING on LPS-induced IFN-β production in mice. (n = 6, *P＜0.05 vs. Control+WT group, #P＜0.05 vs. LPS+WT group).

**Supplementary Fig. 3** CCCP increased the mtDNA level in cytosol of peritoneal macrophages. (n = 6, *P＜0.05 vs. indicated group).

**Supplementary Fig. 4** cGAS knockdown abolished the increase of 2^,^3^,^-cGAMP in peritoneal macrophages induced by LPS. (n = 6, *P＜0.05 vs. indicated group).

**Supplementary Fig. 5** A-B cGAS deficiency inhibited the production of IL-1β and IL-18 in lung tissues. (n = 6, ^&^P＜0.05 vs. Control+WT group, ^$^P＜0.05 vs. LPS+WT group).

**Supplementary Fig. 6** A. The effect of STING overexpression on NLRP3 protein expression in macrophages. B-C. The effects of STING overexpression on the production of IL-1βand IL-18 in peritoneal macrophages. D. The effects of STING overexpression on the level of mtDNA in cytosol of peritoneal macrophages. E. The effect of STING activation on NLRP3 protein expression in macrophages. F-G. The effects of STING activation on the production of IL-1βand IL-18 in peritoneal macrophages. H. The effect of STING activation on the level of mtDNA in cytosol of peritoneal macrophages. (n = 6, *P＜0.05 vs. indicated group).

**Supplementary Fig. 7** TLR4 is essential for the initiation of cGAS-STING pathway triggered by LPS. A. The effects of TLR4 knockdown on the protein levels of cGAS, STING and c-Myc in peritoneal macrophages. B-C. The effect of TLR4 knockdown on the production of IL-1β and IL-18 in peritoneal macrophages. (n = 6, ^*^P＜0.05 vs. PBS group, ^#^P＜0.05 vs. LPS group).


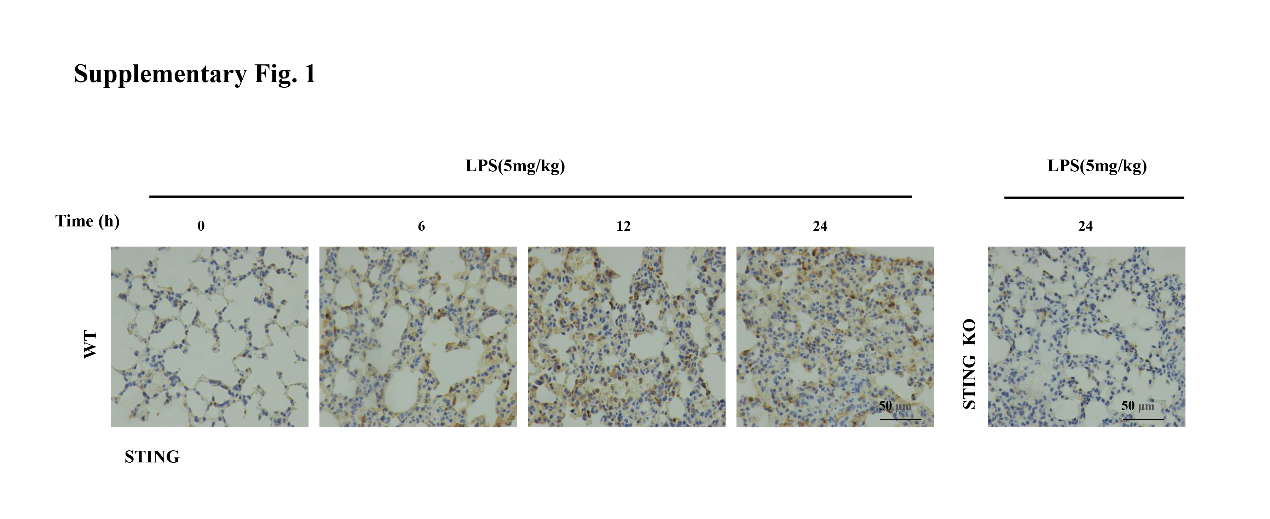

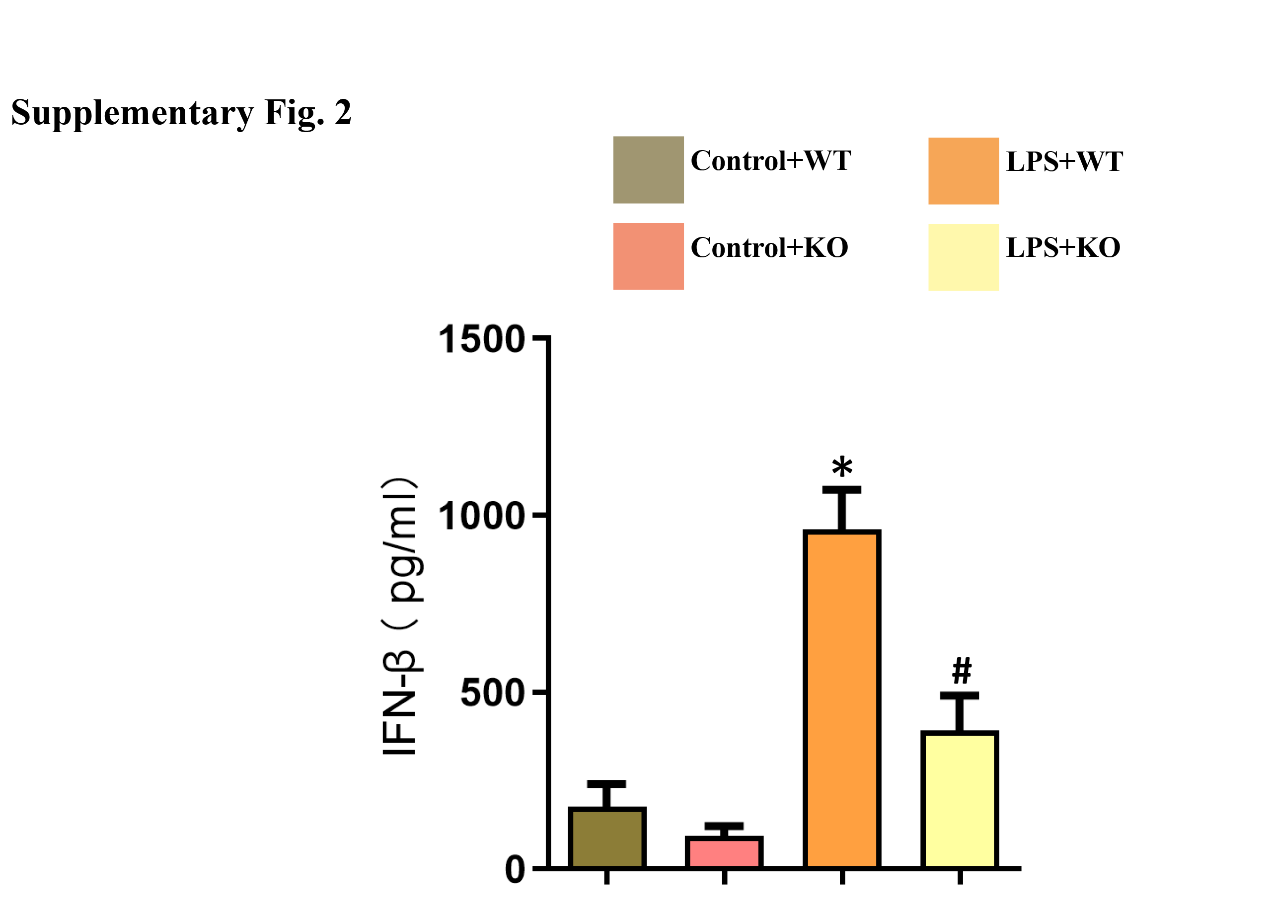


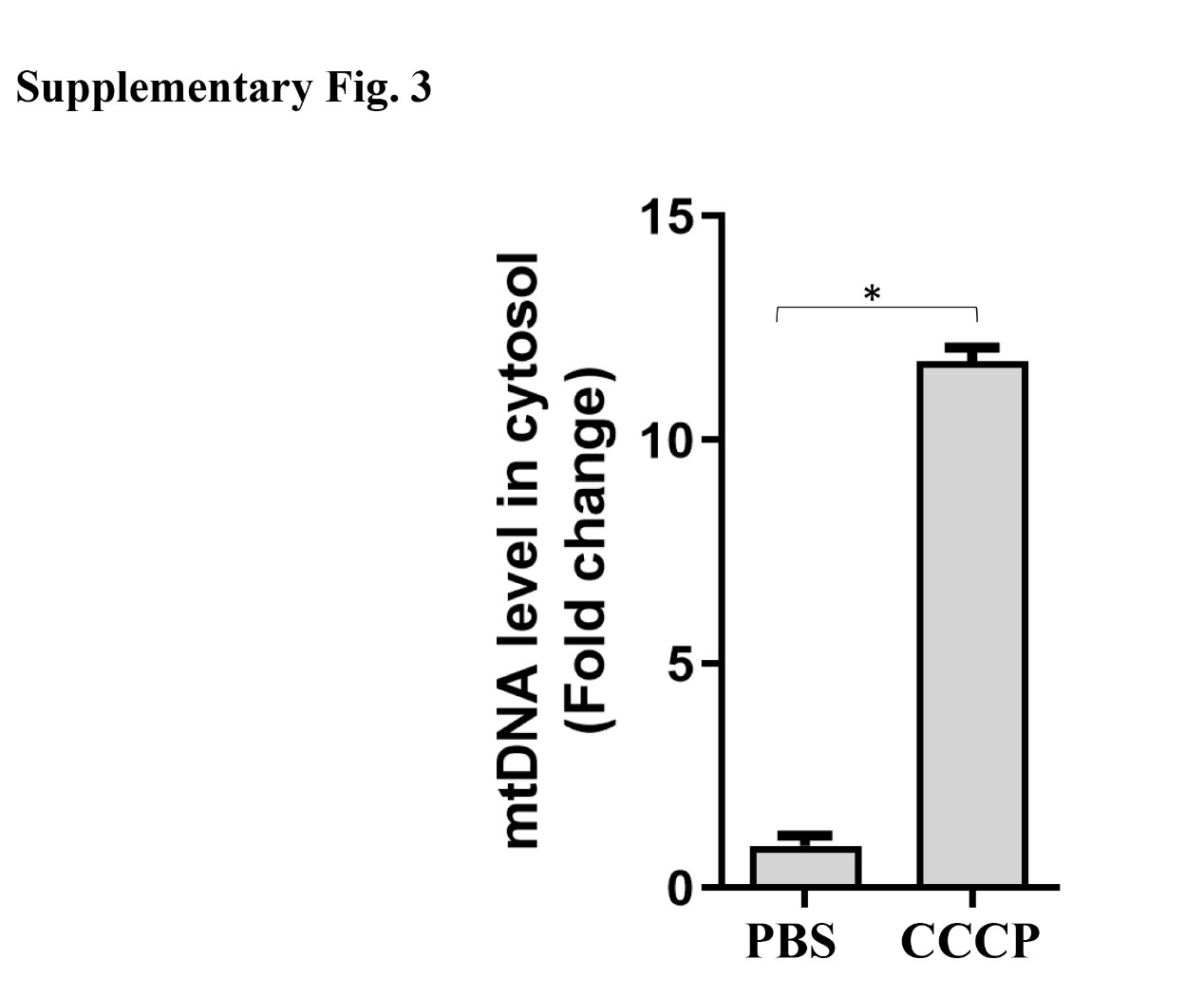


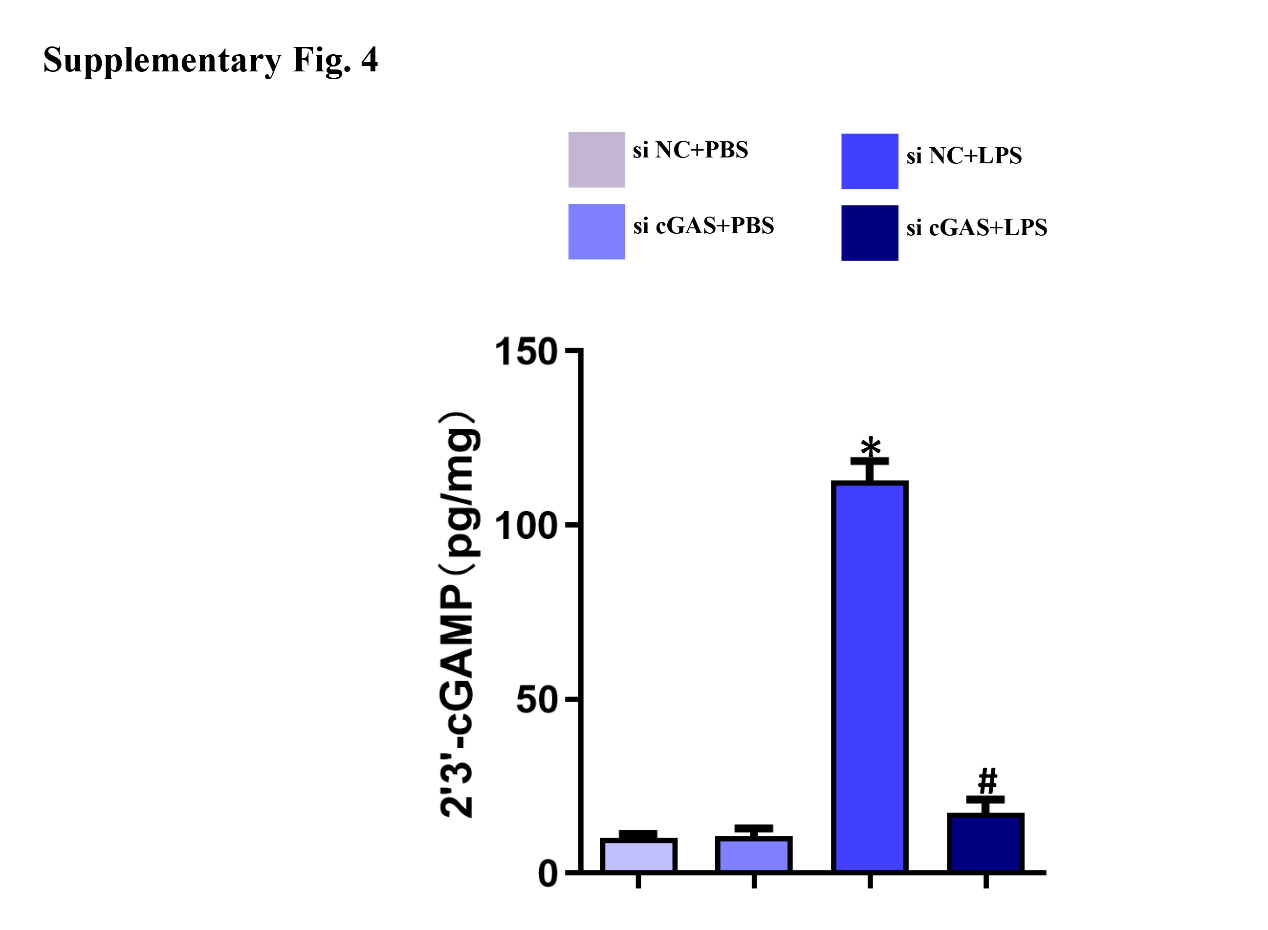


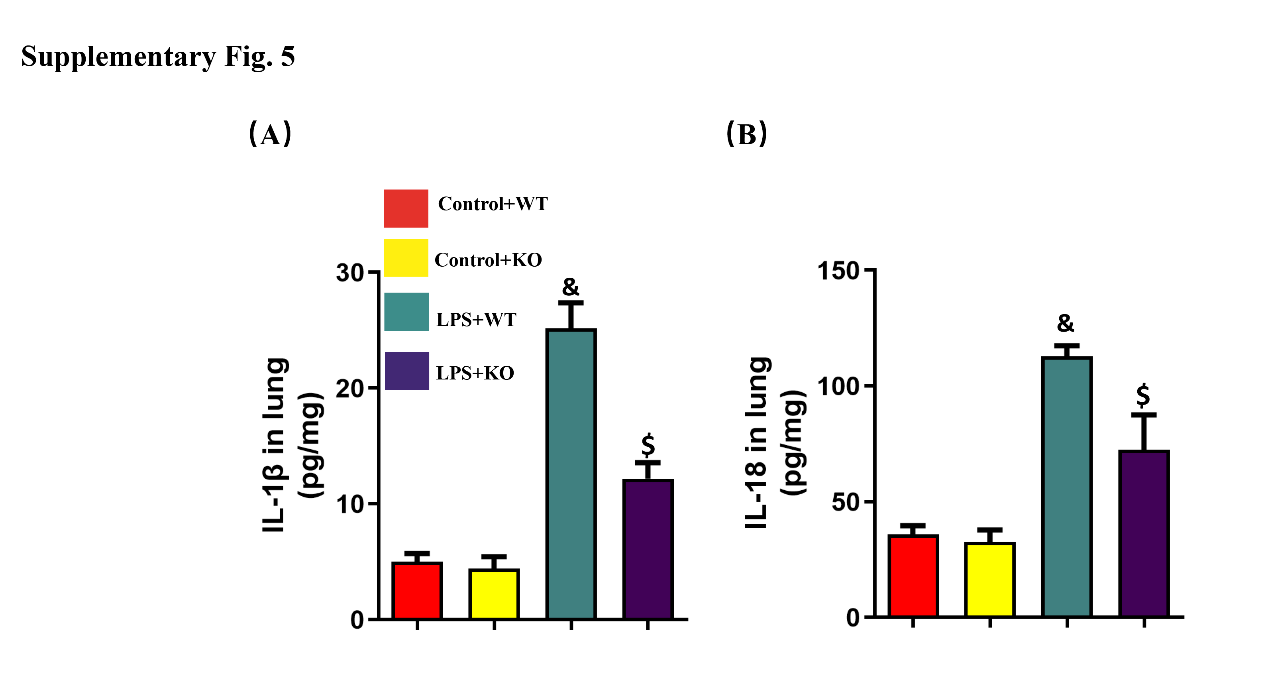


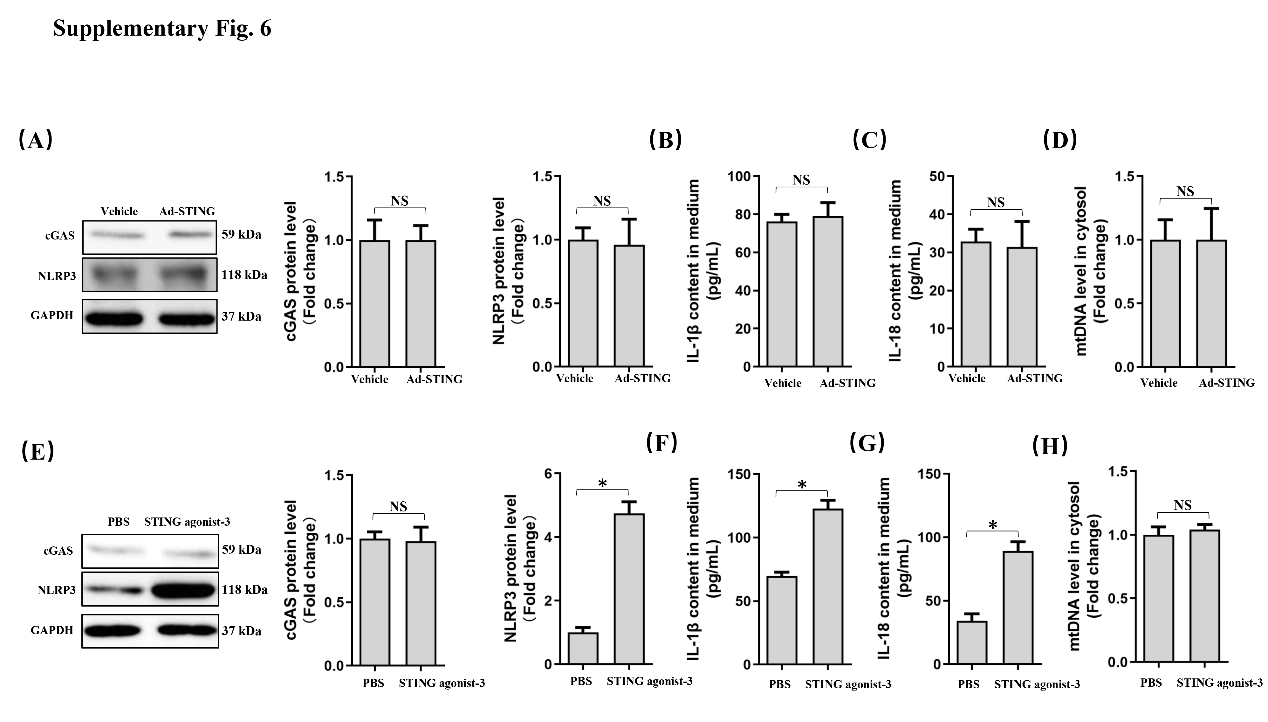


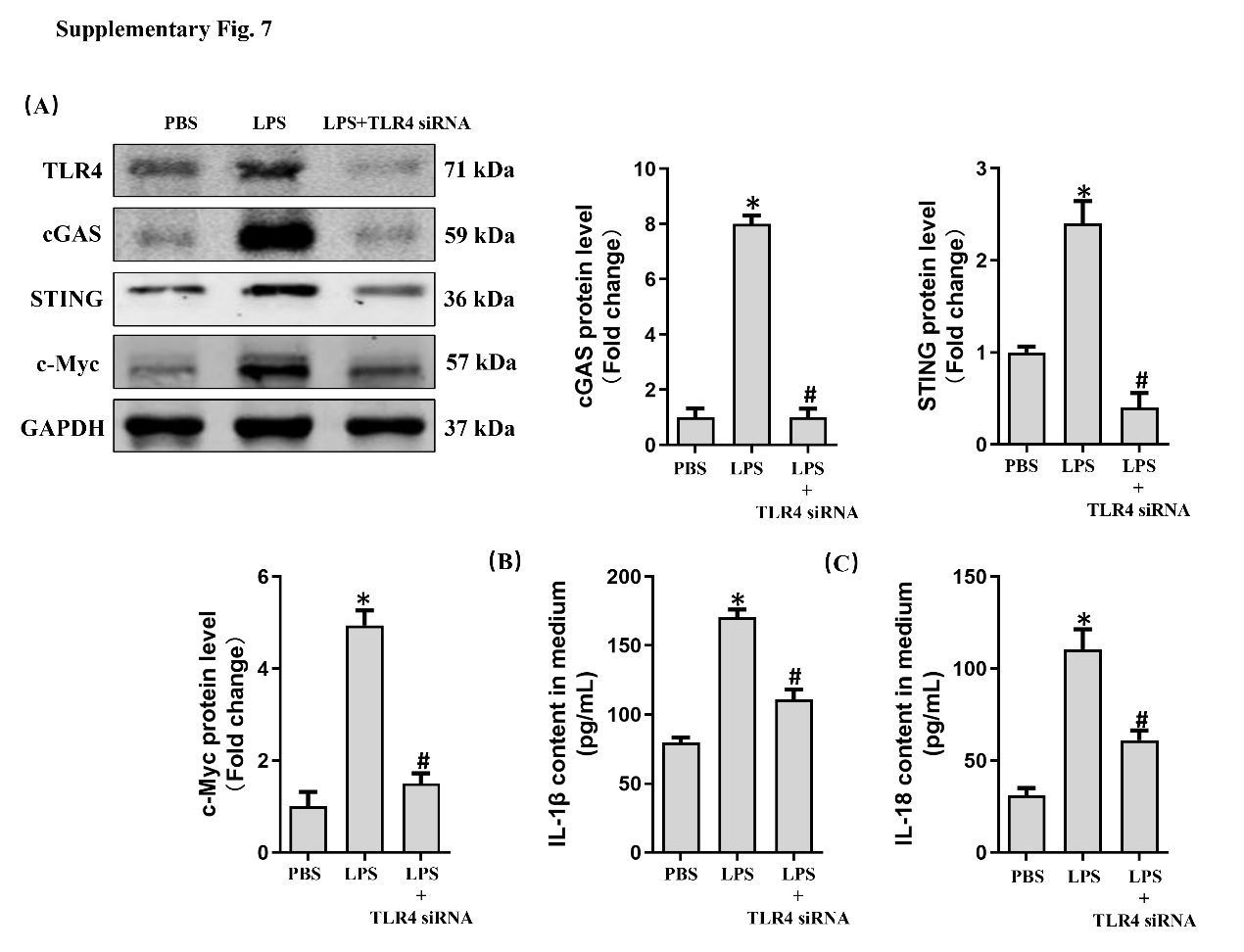

Supplement: Supplementary file 1 — Supplementary Material [file CTM2-10-e228-s001.docx]
